# Adapting gait with asymmetric visual feedback affects deadaptation but not adaptation in healthy young adults

**DOI:** 10.1371/journal.pone.0247706

**Published:** 2021-02-25

**Authors:** Sarah A. Brinkerhoff, Patrick G. Monaghan, Jaimie A. Roper

**Affiliations:** School of Kinesiology, Auburn University, Auburn, Alabama, United States of America; São Paulo State University (UNESP), BRAZIL

## Abstract

Split-belt treadmill walking allows researchers to understand how new gait patterns are acquired. Initially, the belts move at two different speeds, inducing asymmetric step lengths. As people adapt their gait on a split-belt treadmill, left and right step lengths become more symmetric over time. Upon returning to normal walking, step lengths become asymmetric in the opposite direction, indicating deadaptation. Then, upon re-exposure to the split belts, step length asymmetry is less than the asymmetry at the start of the initial exposure, indicating readaptation. Changes in step length symmetry are driven by changes in step timing and step position asymmetry. It is critical to understand what factors can promote step timing and position adaptation and therefore influence step length asymmetry. There is limited research regarding the role of visual feedback to improve gait adaptation. Using visual feedback to promote the adaptation of step timing or position may be useful of understanding temporal or spatial gait impairments. We measured gait adaptation, deadaptation, and readaptation in twenty-nine healthy young adults while they walked on a split-belt treadmill. One group received no feedback while adapting; one group received asymmetric real-time feedback about step timing while adapting; and the last group received asymmetric real-time feedback about step position while adapting. We measured step length difference (non-normalized asymmetry), step timing asymmetry, and step position asymmetry during adaptation, deadaptation, and readaptation on a split-belt treadmill. Regardless of feedback, participants adapted step length difference, indicating that walking with temporal or spatial visual feedback does not interfere with gait adaptation. Compared to the group that received no feedback, the group that received temporal feedback exhibited smaller early deadaptation step position asymmetry (*p* = 0.005). There was no effect of temporal or spatial feedback on step timing. The feedback groups adapted step timing and position similarly to walking without feedback. Future work should investigate whether asymmetric visual feedback also results in typical gait adaptation in populations with altered step timing or position control.

## Introduction

The ability to adjust a walking pattern to meet task demands–gait adaptation–is crucial to mobility [1]. Gait adaptation is commonly assessed on a split-belt treadmill (SBT), which has two independent belts. One measure of SBT gait that adapts is step length asymmetry [2–5]. Initially, healthy individuals walk on the SBT with asymmetric step lengths, where the leg on the slow belt takes a longer step than the leg on the fast belt [2]. Step length asymmetry gradually decreases over the course of ten minutes, despite the belts still moving at different speeds. Provided more than ten minutes to adapt, step lengths become asymmetric such that the fast leg takes a longer step than the slow leg [4] likely in response to the work done by treadmill on the legs [6–8]. Healthy individuals adjust their walking patterns on an SBT by adapting temporal (step timing) and spatial (step position) parameters when gait is perturbed [9, 10]. Here, step timing refers to the time taken between the previous and the current foot strikes, and step position refers to the distance between the pelvis and the lead ankle at foot strike.

When re-exposed to a typical treadmill, on which the belts are moving at the same speed, healthy adults deadapt and return to their normal gait pattern, evidenced by aftereffects in step length, step timing, and step position asymmetries that subsequently return to normal [2, 11, 12]. The aftereffect in step length asymmetry is opposite to the asymmetry seen during initial adaptation. When then exposed to the same SBT perturbation a second time–readaptation–the initial step length asymmetry is less than that seen during adaptation, demonstrating a learning of the new gait pattern [11, 12].

Step timing and position may be dissociable and controlled separately, evidenced by the alteration of spatial gait parameters independent of step timing [13–16]. In fact, cortical activity in felines measured during obstacle crossing indicate that separate cortical areas code for position and timing during movement [17, 18]. Distinct populations of neurons in posterior parietal cortex code for the distance from an object and for the timing before contact with the object [17, 18]. This separation of spatial and temporal information allows for the discrete adjustment of limb movements in both space and in time. Recent mouse [16] and human [13] studies suggest that individuals cannot alter step timing without affecting step position. During SBT walking, individuals provided with visual feedback and targets to walk with symmetric step positions continued to adapt their step timing; conversely, individuals provided with visual feedback and targets to walk with symmetric step times did not adapt step position [13]. Step position may be explicitly controlled without affecting step timing, but explicit control of timing affects step position.

While we know that restricting adaptation in step timing affects position [13], we do not know how providing feedback and targets congruent with step timing adaptation will affect step length adaptation. As healthy individuals adapt to the SBT over ten minutes, they reduce the initial asymmetry in step length [2, 3, 5]. To achieve this initial reduction in step length asymmetry while the belt speeds are different, step timing and position asymmetries increase to combat the difference in belt–therefore, leg–speeds. Long and colleagues reported that healthy adults given spatial feedback do not adapt step length asymmetry or step timing differently than those given no feedback as belt asymmetry was gradually introduced [14]. The effect of step position feedback on non-gradual SBT walking remains unknown. Asymmetric step timing may indeed be an implicit goal of gait adaptation, considering that asymmetric step timing is a determinant of the energy cost of SBT walking [19]. Understanding how the rate and magnitude of step timing and position adaptation can be manipulated during SBT walking is important for studying populations with impaired step timing or position control, such as those with a history of stroke [9, 20], cerebellar ataxia [21], and essential tremor [22].

The purpose of this study was to investigate the effects of temporal and spatial visual feedback and targets of step timing and position asymmetry on 1) step length difference, step timing asymmetry, and step position asymmetry, and 2) rates of adaptation, deadaptation, and readaptation. We asked healthy young adults to adapt on an SBT and step to either asymmetric temporal or spatial feedback congruent with adaptation. We asked a third group to walk on an SBT with no feedback. We measured step length difference, step timing, and step position over a set number of steps in adaptation, deadaptation, and readaptation. Conditions were measured by steps rather than time based on prior research suggesting that one step is considered one trial [23, 24], and to compare our results to those of Gonzalez-Rubio and colleagues that held timing and position symmetric [13]. We measured raw step length difference as opposed to normalized step length asymmetry for consistency with the raw step timing and step position feedback provided to participants.

Considering that step timing affects step position [10, 13–16], we hypothesized that temporal feedback would affect the adaptation, deadaptation, and readaptation of both step timing and step position. Conversely, considering the control of step position does not seem to affect step timing, we hypothesized that spatial feedback would affect the adaptation, deadaptation, and readaptation of step position only. We expected that both feedback groups would adapt step length difference quicker than the group provided no feedback.

## Methods

### Participants

We recruited thirty young adults ages 19 to 35 from the Auburn community. Participants were excluded if they had: loss of vision, peripheral neuropathy, vestibular dysfunction, active unstable medical or psychiatric conditions, diabetes, any orthopedic condition, a history of lower extremity surgery or injury requiring physical therapy, injury to the lower extremity in the last six months, a score of less than 24 out of 30 on the Mini-Mental State Exam [25] (due to the lack of literature citing Mini-Mental State Exam cut-off scores for cognitive impairment in young adults, the established threshold value for older adults was used), or previous experience walking on an SBT with belt speeds decoupled. Participants were also excluded if they took medications affecting balance or alertness/attention (i.e., medications for depression, anxiety, and allergies). Auburn University student participants were offered course extra credit for participating. All participants provided written informed consent before participating in the study as approved by the Auburn University Institutional Review Board. There were always two researchers involved in the data collection [26], and there were up to four other research assistants in the room not observing the data collection but processing data.

Participants were randomized into one of three groups. We performed a power calculation, conservatively estimating an effect size of 0.25 and using an alpha of 0.05, for change in step length difference. Twenty-four participants (6 per group) was needed to detect significant interactions between 3 groups across 6 epochs with a power of 0.80. To be consistent with the number of participants in similar studies, a total of thirty participants were recruited for this study and randomized into the three groups. However, one participant in the Spatial Feedback group screened out due to an inability to step to the spatial targets, resulting in nine participants in the Spatial Feedback group: No Feedback (n = 10), Temporal Feedback (n = 10), and Spatial Feedback (n = 9). Similar sample sizes have been previously studied to explore the effects of spatial and temporal visual feedback on gait adaptation (Gonzalez-Rubio et. al., 2019, n = 7 per group [13]; Long et. al., 2016, n = 10 per group [14]).

### Locomotor adaptation paradigm

Kinematic data were collected from bilateral, passive-reflective markers placed according to the Vicon Nexus Lower-Body Plug-in-Gait Functional Ai model using a 17-camera motion capture system (100 Hz; Vicon, Oxford, UK, version 2.10) while participants walked on an instrumented SBT (1000 Hz; Bertec Corporation, Columbus, OH). Each participant first walked through the lab space to orient the participant to the lab environment. The participant then began the SBT protocol. Participants held on to the side handrails for the duration of the study [11, 22]. They were instructed to look at the display in front of them throughout SBT walking. The electronic display was located directly in front of the participant at eye level for all SBT walking conditions. Considering the effect of instruction on gait, participants were all provided the same instructions [27].

First, all groups warmed up for two minutes by walking with the belts tied at 0.75 m/s. Then, participants were given two minutes to practice the visual feedback paradigm while the belts were tied at 0.75 m/s. Two minutes was sufficient for participants to reach 100 steps, which has been previously used to familiarize participants to a visuomotor walking task [28]. No participant needed longer than two minutes to become familiar with the paradigm. More details on the visual targets are provided in the *Visual Feedback Paradigm* section. After the two-minute visual feedback practice, the belts were tied at 1.0 m/s for 150 strides (about three minutes) then tied at 0.5 m/s for 150 strides (baseline, about 5 minutes), during which the screen displayed fixation crosses for all groups. We defined a stride as heel-strike to heel-strike of the same limb.

After baseline walking at 0.5 m/s, the treadmill was stopped completely. Then the treadmill belts were set to the speeds for the “split” condition (adaptation). To ensure that during adaptation step length difference error would be augmented, the assigned slow leg was the leg that had a longer step length during the last three strides of the tied walking warmup at 0.75 m/s. Sixteen of twenty-nine participants (55%) walked with their dominant leg taking a longer step. While walking at 0.75 m/s, participants walked with 19.3 ± 13.1 mm of step length difference, which was an asymmetry of 2.0 ± 1.4% of participant stride length. The fast belt was set to 1.0 m/s and the slow belt was set to 0.5 m/s. This adaptation condition was maintained for 600 strides (about 14 minutes), during which the Feedback groups were given visual temporal or spatial feedback and the No Feedback group was given fixation crosses. The treadmill was stopped completely following adaptation.

Then, the treadmill belts were tied at 0.5 m/s for 150 strides (deadaptation, about 5 minutes) to test for aftereffects, during which the screen displayed fixation crosses for all groups. The treadmill was again stopped completely. Following deadaptation, the treadmill belts were set to the same split condition as in adaptation for 150 strides (readaptation, about 3.5 minutes); the fast belt was set to 1.0 m/s, and the slow belt was set to 0.5 m/s, during which the screen displayed fixation crosses for all groups.

### Visual feedback paradigm

#### No Feedback group

**Fig 1:**
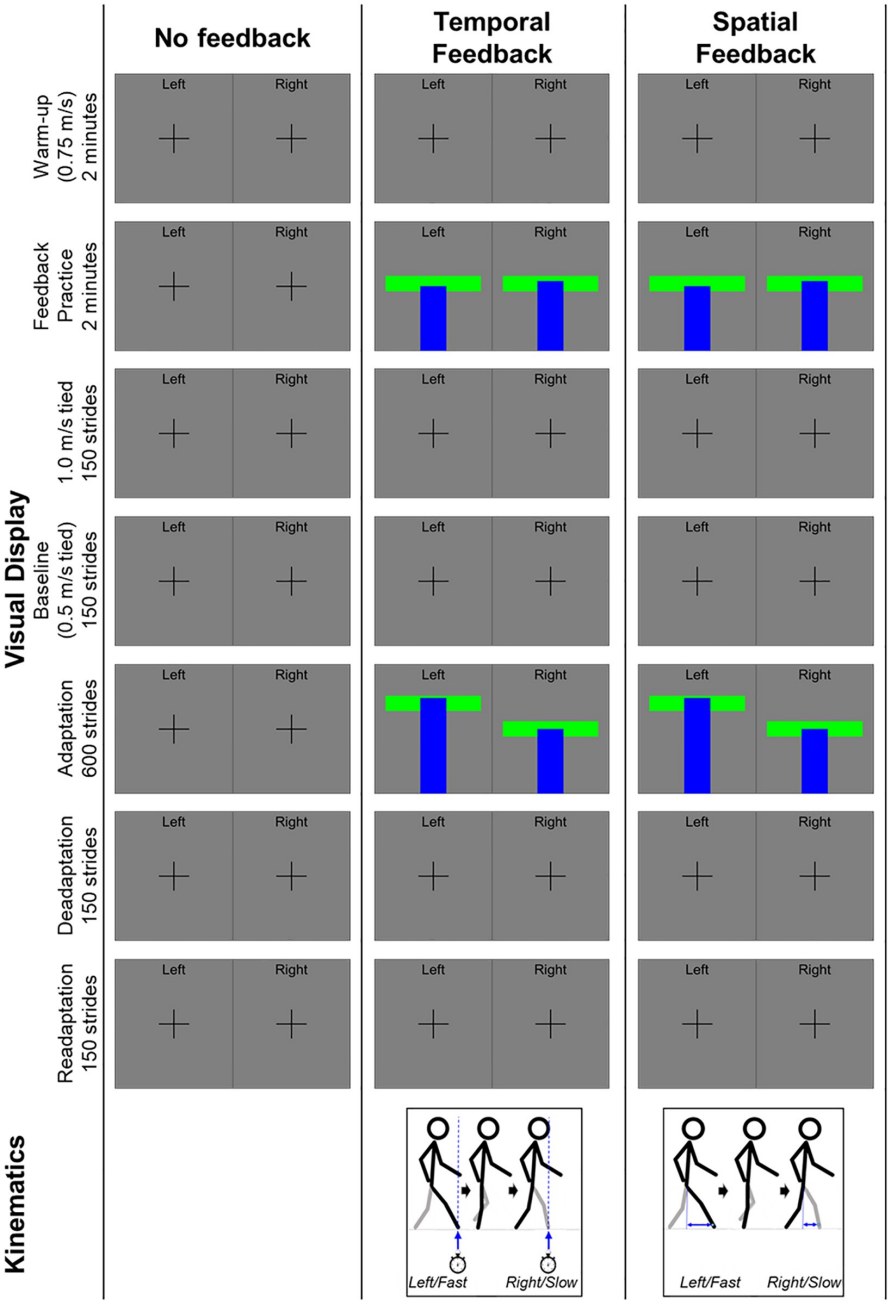
Example television display and depiction of the kinematics given as feedback. The display was split into left and right, representative of the left and right steps. The No Feedback group saw fixation crosses for all walking conditions. Temporal and Spatial Feedback groups saw targets and feedback for the two-minute practice condition and the adaptation condition and saw fixation crosses for all other conditions. The feedback for both Feedback groups looked the same: horizontal target boxes for each leg with vertical blue bars that changed height with each step based on real-time kinematics. If the top of the blue bar landed within the target box, the target box turned green. If the top of the blue bar landed above or below the target box, the target box turned red. In this example, the fast leg–determined prior to the adaptation condition–is the left leg for the participant in both the Temporal and Spatial Feedback groups.

The No Feedback group saw fixation crosses displayed on the screen on both the left and right sides for all conditions–two-minute warmup, two-minute visual feedback practice, 1.0 m/s walking, baseline 0.5 m/s walking, adaptation, deadaptation, and readaptation (Fig 1). Participants in the No Feedback group were provided the following fixation cross instructions for each walking condition: “Look at the screen in front of you and focus on the crosses as you walk. Look at the left cross as you take a left step and look at the right cross as you take a right step.”

#### Feedback groups

Participants in both the Temporal and the Spatial Feedback groups saw fixation crosses and received the fixation cross instructions during the 0.75 m/s walking warmup (Fig 1). During the two-minute visual feedback practice, participants in the Feedback groups saw targets and real-time temporal or spatial visual feedback. The targets presented to the Feedback groups during feedback practice were symmetric and were set to the participant’s recorded natural step timing (Temporal Feedback group) or step position (Spatial Feedback group). The targets for feedback practice were calculated from the last three strides [29, 30] of the 0.75 m/s tied walking warmup. The Feedback groups were instructed to walk by keeping their step timing or position within the targets. Then, during 1.0 m/s and 0.5 m/s tied walking, participants in the Feedback groups saw fixation crosses on the screen.

During adaptation, participants in the Feedback groups again saw targets and real-time temporal or spatial feedback, calculated from the last three strides during each of the 1.0 m/s and 0.5 m/s tied walking conditions. Therefore, the horizontal target boxes were asymmetric. The targets were intended to simulate the step timing and step position the participant would achieve without feedback by the end of 1200 steps. Considering that individuals would achieve different step timing and step position values by the end of 1200 steps, the targets chosen were reflective of each individual’s natural stepping pattern at each belt speed when the belts were tied. Pilot testing demonstrated that participants could physically achieve the step timing or position asymmetries calculated from tied-belt walking, and that these asymmetries were consistently significantly asymmetric across participants. The fast leg always targeted the higher absolute value of step timing or step position, as this is how adaptation occurs in both the temporal and the spatial domain when no visual feedback is present. Therefore, for the Temporal Feedback group, the fast leg targeted the step timing when walking tied at 0.5 m/s (longer time), and the slow leg targeted the step timing when walking tied at 1.0 m/s. For the Spatial Feedback group, the fast leg targeted the step position when walking tied at 1.0 m/s (more anterior foot placement), and the slow leg targeted the step position when walking tied at 0.5 m/s. Finally, during both deadaptation and readaptation, participants in the Feedback groups saw fixation crosses on the screen.

#### Description of visual targets and feedback

When targets and real-time feedback were displayed, the left and right targets were depicted as horizontal boxes, and the real-time step feedback was depicted as left and right vertical blue bars. Consistent with the measured outcomes, temporal targets and feedback were measured as the time between consecutive contralateral foot strikes. Spatial targets and feedback were measured as the anteroposterior distance from the ankle marker to the respective anterior pelvis marker at foot strike. With each step, the vertical blue bars changed size based on either when or where the participant stepped for the Temporal or Spatial Feedback group, respectively. When targets and feedback were on the screen, participants in the Temporal Feedback group were instructed, “Look at the screen in front of you. Aim to match the size of the bar to the target area on the screen, for each leg, by adjusting when, not where you step down on the treadmill.” Participants in the Spatial Feedback group were instructed, “Look at the screen in front of you. Aim to match the size of the bar to the target area on the screen, for each leg, by adjusting where, not when you step down on the treadmill.” The horizontal target box turned green when participants stepped within the target and turned red when participants stepped outside of the target.

The maximum display height was calculated as the average of the two targets multiplied by 1.75 (Temporal Feedback) or 2.75 (Spatial Feedback). The scaling factors were chosen empirically from pilot testing as the optimal gain for successful targeting while ensuring that the target boxes did not overlap. The error tolerance was determined as ±5% of the display height such that the target area was a consistent size for both legs and for all participants (average Temporal Feedback target height: 0.13 sec ± 0.01 sec; average Spatial Feedback target height: 35 mm ± 4 mm). The error tolerance relative to target values was 9 ± 2% for the Temporal Feedback group and 15 ± 4% for the Spatial Feedback group. If a participant’s step landed outside of the target, that step was counted as a missed step and the target box turned red.

### Data analysis

Treadmill gait events were marked in real-time using force data, for use in the visual feedback paradigms and counting steps during each trial. The force data were not filtered live during data collections, so a force threshold was empirically found to mark gait events consistently and accurately above the noise of the treadmill (80 N threshold). Gait events were marked post-processing by a custom MATLAB code (Mathworks, Natick, USA, version 2020a) using unfiltered force data with the same threshold as the real-time event marking so that the analysis would reflect the real-time step timing and position feedback. If a participant crossed over the midline of the treadmill, kinematic data were used to mark gait events for the respective step.

Step length was calculated as the anteroposterior distance between the ankle markers at foot strike. Step timing was calculated as the time between contralateral consecutive foot strikes where *time*_*slow leg*_ was the time, in seconds, from a fast leg foot strike to the subsequent slow leg foot strike. Step position was defined as the anteroposterior distance from the ankle marker to the respective anterior pelvis marker at foot strike. These calculated variables were the same as the real-time targets and feedback to participants. These parameters have been used previously as both feedback and measurement [13]. Asymmetry (step length, step timing, and step position) was calculated using the following equation.

Asymmetry=fastleg-slowleg

An asymmetry value of zero indicates that the legs on the fast and the slow belts were symmetric in the respective outcome measure. A positive asymmetry score indicates that the leg on the fast belt had a higher value (longer step length, longer time to step, or stepped further in front of the pelvis) than the leg on the slow belt. Step length difference, step timing asymmetry, and step position asymmetry were measured during six epochs: early adaptation, early deadaptation, and early readaptation (first five strides) and at the plateau (mean of the last 30 strides) of adaptation, deadaptation, and readaptation [20, 31].

The rates of adaptation in step length difference, step timing asymmetry, and step position asymmetry were inferred from the number of steps a participant took until five consecutive strides were within two standard deviations of the plateau during adaptation, deadaptation, and readaptation. Although steps to plateau is not a direct measurement of the rate of adaptation, it is indicative of the rate, where a larger number of steps to reach a plateau is a lower rate of adaptation.

Participants walked for 600 strides during adaptation, 150 strides during deadaptation, and 150 strides during readaptation. However, only steady-state gait was analyzed, and data during belt acceleration were not used. Therefore, participants walked for: adaptation, 598 ± 2 strides; deadaptation, 146 ± 5 strides; readaptation, 149 ± 1 strides.

### Statistical analysis

Separate one-way ANOVAs compared group differences in age, height, mass, and typical treadmill walking speed. A three (group) by six (epoch: early and plateau of adaptation, deadaptation, and readaptation) multivariate mixed model ANOVA assessed differences in step length difference, step timing asymmetry, and step position asymmetry. A three (group) by three (condition: adaptation, deadaptation, and readaptation) multivariate mixed model ANOVA assessed differences in the number of steps to plateau in step length difference, step timing asymmetry, and step position asymmetry. When sphericity was violated, a Greenhouse-Geisser correction was used. Statistical significance was set at α = 0.05 for all analyses, and Bonferroni post-hoc adjustments were applied when appropriate.

## Results

One-way ANOVAs revealed no significant group differences in age (*F*(2,26) = 1.599, *p* = 0.221), height (*F*(2,26) = 1.076, *p* = 0.356), mass (*F*(2,26) = 0.145, *p* = 0.866), or typical treadmill walking speed (*F*(2,24) = 0.230, *p* = 0.796) (Table 1). Table 2 provides the *F* statistics, p-values, and effect sizes for the omnibus statistical tests. Participants in the Temporal Feedback group stepped within the targets for 1032 of the 1200 steps, or 86% of the trial (SD: 103 steps, 9%). Participants in the Spatial Feedback group stepped within the targets for 919 of the 1200 steps, or 77% of the trial (SD: 87 steps, 7%).

**Table 1:** Participant demographics in means and (standard deviations).

Group	N	Age (yr)	Height (cm)	Mass (kg)	Sex	Mini-Mental State Exam score	Typical Speed (m/s)	Longer Step at Baseline
No Feedback	10	21 (1)	169.9 (9.7)	75.1 (18.8)	4 M	29 (1)	1.03 (0.13)	6 Right leg
Temporal Feedback	10	20 (1)	173.4 (7.5)	78.0 (15.9)	4 M	29 (1)	1.07 (0.13)	5 Right leg
Spatial Feedback	9	21 (2)	168.0 (7.1)	74.1 (14.9)	3 M	29 (1)	1.04 (0.09)	4 Right leg

Longer Step at Baseline = the leg that naturally exhibited a larger step length when walking at 0.75 m/s tied, which was the leg on the slow belt during adaptation and readaptation.

**Table 2:** Main effects and interactions of the analysis of variances.

Summary of Mixed Model 3 x 6 Multivariate ANOVA for Magnitude of Step Length Difference, Step Position Asymmetry, and Step Timing Asymmetry			
	Group	Epoch F(15,353.753) = 129.995, <**0.001**	Epoch × Group F(30,376.381) = 1.840, **0.005**
Step Length Difference	*F*(2,26) = 0.188, 0.829	*F*(2.798,72.751) = 248.918, **<0.001**	*F*(5.596,72.751) = 2.518, **0.032**
	*η*^*2*^_*p*_ = 0.014	*η*^*2*^_*p*_ = *0*.*905*	*η*^*2*^_*p*_ = *0*.*162*
Step Position Asymmetry	*F*(2,26) = 0.694, 0.508	*F*(2.900,75.388) = 22.922, **<0.001**	*F*(5.799,75.388) = 2.722, **0.020**
	*η*^*2*^_*p*_ = 0.051	*η*^*2*^_*p*_ = *0*.*469*	*η*^*2*^_*p*_ = *0*.*173*
Step Timing Asymmetry	*F*(2,26) = 1.672, 0.207	*F*(2.171,56.449) = 35.389, **<0.001**	*F*(4.342,56.449) = 1.439, 0.230
	*η*^*2*^_*p*_ = 0.114	*η*^*2*^_*p*_ = *0*.*576*	*η*^*2*^_*p*_ = 0.100
Summary of Mixed Model 3 x 3 Multivariate ANOVA for Steps to Plateau in Step Length Difference, Step Position Asymmetry, and Step Timing Asymmetry			
	Group	Condition F(6,100) = 5.824, **<0.001**	Condition × Group F(12,132.579) = 1.100, 0.365
Step Length Difference	*F*(2,26) = 0.834, 0.446	*F*(1.428,37.131) = 13.105, **<0.001**	*F*(2.856,37.131) = 1.760, 0.174
	*η*^*2*^_*p*_ = 0.060	*η*^*2*^_*p*_ = *0*.*335*	*η*^*2*^_*p*_ = 0.119
Step Position Asymmetry	*F*(2,26) = 0.523, 0.599	*F*(1.366,35.510) = 4.255, **0.035**	*F*(2.732, 35.510) = 1.428, 0.252
	*η*^*2*^_*p*_ = 0.039	*η*^*2*^_*p*_ = *0*.*141*	*η*^*2*^_*p*_ = 0.099
Step Timing Asymmetry	*F*(2,26) = 0.619, 0.546	*F*(1.093,28.429) = 9.715, **0.003**	*F*(2.187, 28.429) = 0.173, 0.859
	*η*^*2*^_*p*_ = 0.045	*η*^*2*^_*p*_ = *0*.*272*	*η*^*2*^_*p*_ = 0.013

Data are presented as *F*(df1,df2) = *F*-value, *p*-value. Boldfaced *p*-values indicate statistical significance at the multivariate or univariate follow-up level. Effect size (η^2^_p_) is reported for each effect.

**Fig 2:**
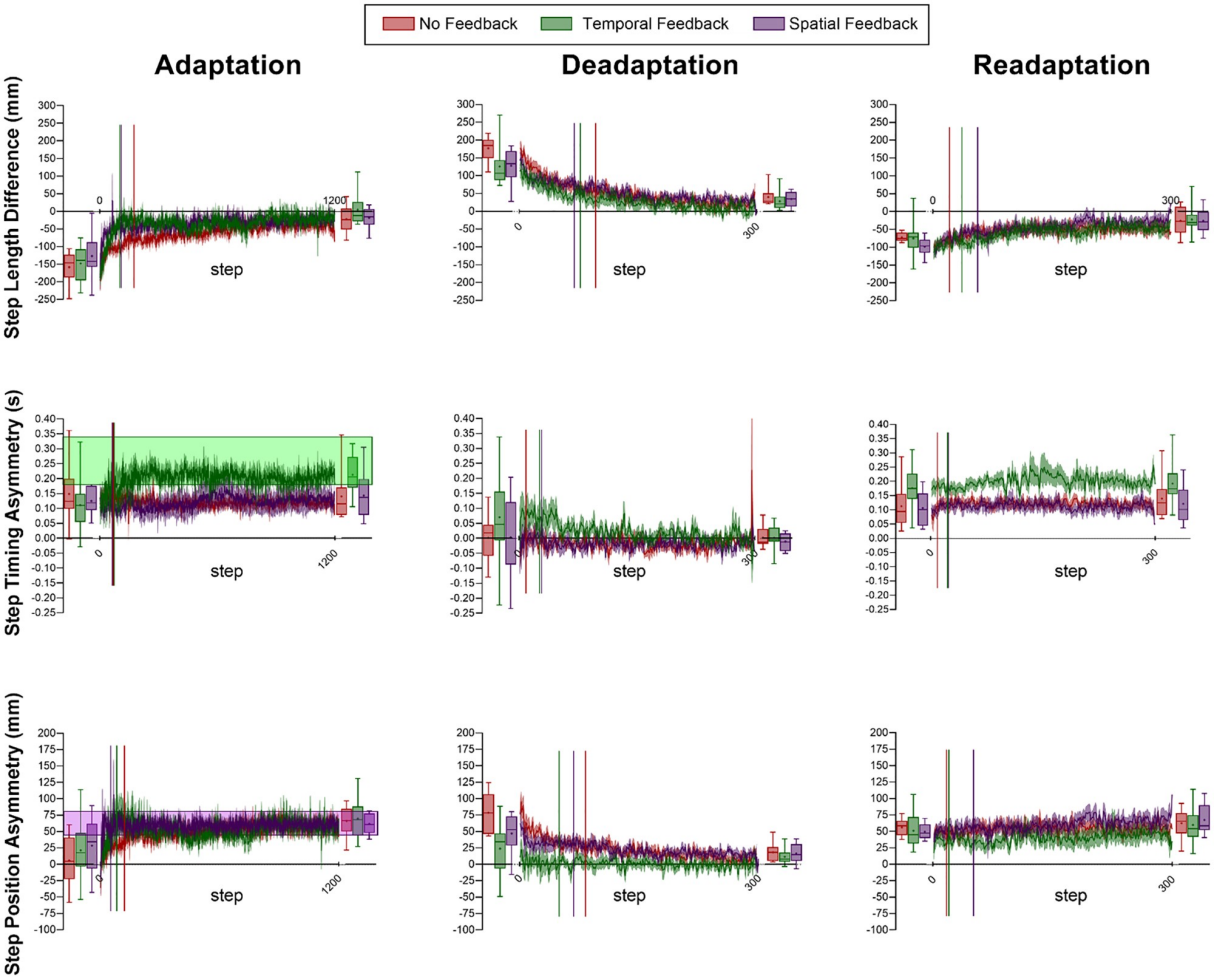
Step length difference, step timing asymmetry, and step position asymmetry during adaptation, deadaptation, and readaptation. Red lines indicate the No Feedback group, green lines indicate the Temporal Feedback group, and purple lines indicate the Spatial Feedback Group. Shaded areas surrounding lines indicate SEM. Box plots before and after the line graphs indicate early and plateau values for each group, respectively, where lines indicate the medians and the upper and lower quartiles, crosshairs indicate the means, and whiskers indicate the minimum and maximum. Shaded rectangles in Adaptation Step Timing Asymmetry and Adaptation Step Position Asymmetry indicate the targeted asymmetry for step timing and step position, respectively.

**Fig 3:**
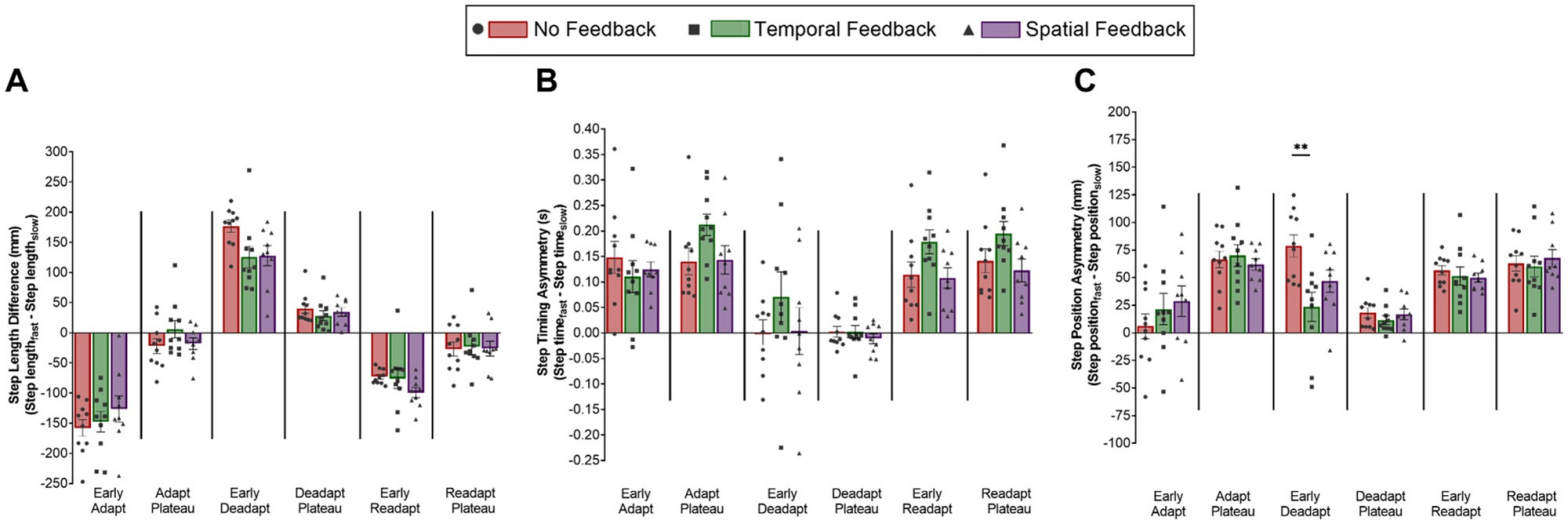
Bar graphs and individual values for A) step length difference, B) step timing asymmetry, and C) step position asymmetry during adaptation, deadaptation, and readaptation. Red bars (circles) indicate the No Feedback group, green bars (squares) indicate the Temporal Feedback group, and purple bars (triangles) indicate the Spatial Feedback Group. Individual points are plotted for all. Error bars indicate SEM. Asterisks indicate condition differences, **p<0.*01*.

Fig 2 depicts the means of each group’s adaptation, deadaptation, and readaptation curves for step length difference, step timing asymmetry, and step position asymmetry. Fig 3 depicts the mean and standard errors of each group’s asymmetry variables in each condition and significant differences. There was a significant multivariate interaction between group and epoch for the magnitude of step length difference, step timing asymmetry, and step position asymmetry. Univariate tests revealed significant interactions between group and epoch in step length difference, in step position, but not in step timing (Fig 3A–3C). In follow-up Bonferroni t-tests, there were no differences between groups within conditions in step length difference (Fig 3A). Follow-up Bonferroni t-tests revealed that the Temporal Feedback group had smaller aftereffects in step position asymmetry–i.e., a smaller magnitude in early deadaptation–than the No Feedback group (*p* = 0.005, d = 1.49, Fig 3C). Otherwise, there were no significant differences between groups within conditions in step position asymmetry.

**Fig 4:**
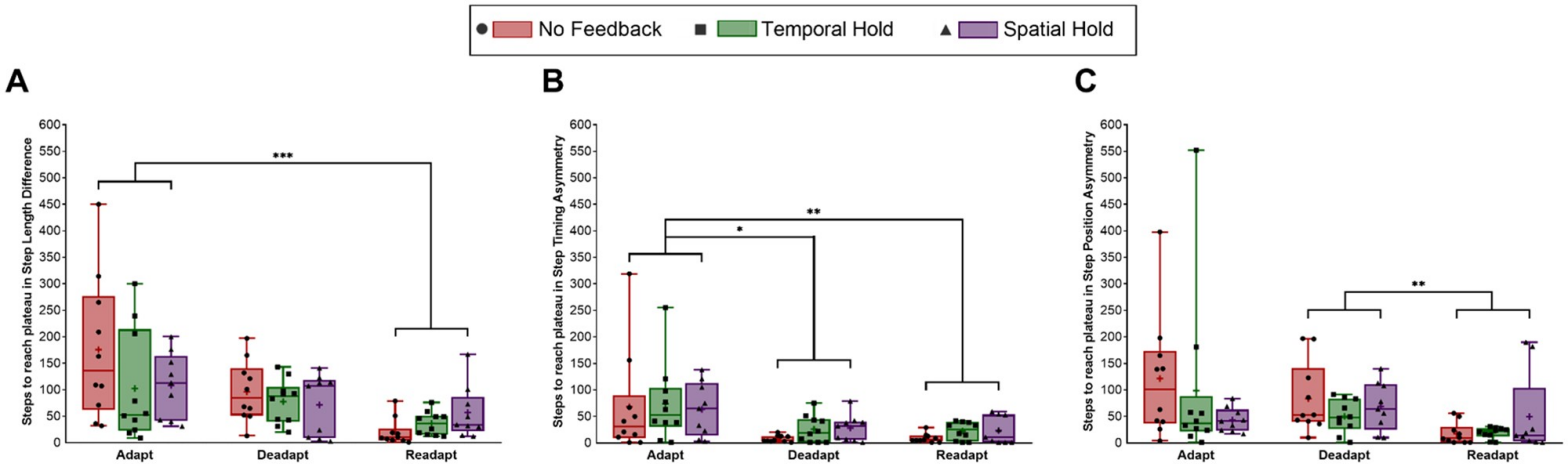
The number of steps it took to reach a plateau in of A) step length difference, B) step timing asymmetry, and C) step position asymmetry during adaptation, deadaptation, and readaptation. Red boxes (circles) indicate the No Feedback group, green boxes (squares) indicate the Temporal Feedback group, and purple boxes (triangles) indicate the Spatial Feedback Group. Lines indicate the medians and the upper and lower quartiles, crosshairs indicate the means, and whiskers indicate the minimum and maximum. Individual points are plotted for all. Asterisks indicate condition differences, *p<0.*05*, **p<0.*01*, ***p<0.*001*.

Fig 4 depicts the mean, median, and quartiles of each group’s adaptation, deadaptation, and readaptation steps to plateau in step length difference, step timing asymmetry, and step position asymmetry, along with significant differences. There was a significant multivariate effect of condition on the number of steps to plateau, but no multivariate interaction. There were significant main effects of condition in all three variables. Participants reached a plateau in step length difference in fewer steps (higher rate) during readaptation than during adaptation (*p* < 0.001, d = 1.09, Fig 4A). Participants reached a plateau in step timing in fewer steps (higher rate) during deadaptation than during adaptation (*p* = 0.015, d = 0.84), and in fewer steps (higher rate) during readaptation than during adaptation (*p* = 0.009, d = 0.89, Fig 4B). Participants reached a plateau in step position in fewer steps (higher rate) during readaptation than during deadaptation (*p* = 0.008, d = 0.91, Fig 4C).

## Discussion

The purpose of this study was to investigate the effects of asymmetric step timing and step position visual feedback and targets on 1) step length difference, step timing asymmetry, and step position asymmetry, and 2) rates of adaptation, deadaptation, and readaptation. This study has four main findings. First, regardless of group, all participants were able to adapt to the perturbation induced by the SBT. Second, we hypothesized that visual feedback would result in faster step length difference adaptation–however, visual feedback did not affect the rate of adaptation. Third, we hypothesized that temporal feedback would affect step timing–however, neither temporal nor spatial feedback altered step timing. Fourth, we hypothesized that both temporal and spatial feedback would affect step position–while temporal feedback altered step position deadaptation, spatial feedback did not alter step position.

Despite a group difference in step position aftereffects, all groups responded typically to the SBT; step length difference decreased throughout adaptation, aftereffects in step length difference decreased throughout deadaptation, and step length difference was smaller upon second exposure to the SBT. Therefore, stepping to explicit visual feedback did not change overall gait adaptation, deadaptation, or readaptation. In line with previous literature, healthy young adults walking with congruent visual feedback adapt the magnitude of step length difference similar to adaptation without feedback [14]. We did observe smaller aftereffects in step position in the Temporal Feedback group. Interestingly, these aftereffects did not carry over into aftereffects in step length difference. However, our measurement of step timing and position should not sum to step length difference when foot velocity is included, as should occur if we had measured temporal, spatial, and velocity contributions to step length difference [9]. Notably, we specifically measured the timing between steps, and did not incorporate foot velocity into the timing parameter. We also did not include measurement of the velocity contribution to step length difference, which is largely indicative of the belt speed differences [9]. Therefore, it is not incongruous to observe aftereffects in step position but not in step length difference.

We did not observe group differences in step length difference, step timing asymmetry, nor step position asymmetry during adaptation. Although we interpreted this finding to mean that the feedback provided resulted in gait adaptation similar to adaptation without feedback, it is possible that participants simply ignored the feedback and instead adapted normally to the SBT. However, we found that healthy young adults can respond to this feedback during gait adaptation considering two key results. First, participants reported explicitly modifying their step timing or step position based on the feedback and targets provided and reported engagement in the feedback for the duration of the SBT condition. Second, plateau values were not different across groups indicating that step timing and position plateau values were correctly calculated, and participants were able to achieve those values while stepping on-target for 86% (Temporal Feedback) and 77% (Spatial Feedback) of the total steps. Despite our confidence in the validity of the results of the visual feedback, future work should consider alternative hypotheses, such as participants ignoring the feedback. Future work should replicate these results in healthy young adults at different belt speed ratios (e.g., 3:1) and magnitudes (e.g., 1.5 and 0.75 m/s) to determine if the current visual feedback would induce different spatiotemporal stepping strategies during larger perturbations.

Considering that adaptation did not differ due to temporal or spatial feedback, healthy individuals may continue to use proprioceptive feedback from the treadmill even when explicit visual feedback is provided. Populations with impaired proprioception or altered spatiotemporal mechanics may benefit from visual feedback during gait adaptation. For example, people with Parkinson’s disease demonstrate impairment in the proprioceptive system [32, 33], and people with essential tremor demonstrate impairment in temporal gait adaptation [22]. In such populations with existing impairments, visual feedback during gait adaptation may be beneficial. In fact, visual feedback is known to improve gait in people with Parkinson’s disease (see Muthukrishnan et. al. for a review [34]). Future work should study the efficacy of visual feedback on gait adaptation in populations with impaired proprioception or altered spatiotemporal mechanics, such as people with Parkinson’s disease [32, 33], moderate to severe cerebellar ataxia [21], a history of stroke [9, 20], and essential tremor [22].

There was no difference in the number of steps to plateau between groups for step length difference, step timing asymmetry, or step position asymmetry during adaptation, deadaptation, or readaptation. We sampled healthy young adults that adapted step length difference quickly–on average, in 65 ± 54 strides. This rate of adaptation is comparable to that observed in previous studies. Although Roemmich et. al. [31] did not measure rate of adaptation, adaptation in the feedback group was deemed faster than in the no-feedback group due to a smaller step length difference over the first 6–200 steps (~3–100 strides). Conversely, Malone and Bastian reported that step length difference and spatial control adapted within about 200 strides in a no-feedback group, while temporal control adapted within about 100 strides [15]. These values are about three times the observed rate in the current study; however, it is imperative to note methodological differences. The current study determined that a plateau was attained when the magnitude of the variable in question was within two standard deviations of the plateau, whereas Malone and Bastian determined a plateau was attained when the value was within one standard deviation of the plateau. Additionally, we augmented participants’ baseline asymmetry, which may have led to higher early step length difference than if we had not augmented baseline error. A higher initial magnitude of asymmetry likely requires more steps to reach a plateau than does a lower initial asymmetry.

Neither type of visual feedback affected step timing. The lack of difference in step timing asymmetry between groups during early adaptation indicates that when given no feedback and when given spatial feedback, healthy young adults adjust their step timing relatively quickly, no differently than if given temporal feedback. Likewise, we did not observe differences due to temporal feedback in the aftereffects or readaptation in step timing. The finding that spatial feedback does not affect the adaptation of step timing replicates findings by prior studies [13, 14], adding to the assertion that adaptation of step position can be controlled without affecting the adaptation of step timing.

Our robust effect of the manipulation of step timing on step position is notable and reinforces two recent studies [13, 16]. Specifically, during early deadaptation the effect size in step position between the No Feedback and Temporal Feedback groups was 1.49, indicating that the two groups differed by almost one and a half standard deviations. Therefore, there is a strong effect of temporal feedback during adaptation on step position during deadaptation. Darmohray and colleagues lesioned the interpose cerebellar nuclei in rats that subsequently walked on an SBT [16]. In those rats, temporal gait adaptation was reduced and spatial gait deadaptation was reduced. In both the current study and that from Darmohray and colleagues, manipulation of temporal features during adaptation led to altered spatial features during deadaptation. Gonzalez-Rubio and colleagues studied human gait adaptation using a visual feedback similar to the one in the current study but provided symmetric visual targets to restrict step timing or position adaptation [13]. When step timing was restricted from adapting, step position adaptation was also reduced, again exemplifying the effect on spatial gait features of temporal manipulation [13]. Taken together, these prior studies and the current study suggest that manipulation of temporal gait adaptation affects spatial locomotor learning, but spatial adaptation may be independently manipulated [10, 14].

Given that step position can be directly mapped to physical space and timing cannot, the visual feedback differences should be considered. Participants demonstrate negative asynchrony and high variability in step timing when walking to a visual metronome [35]. Step timing is also made more complex in that it can be manipulated by altering the timing of either stance or swing phase of the stepping leg. Participants may have implicitly retained spatial adaptation when provided explicit temporal feedback. Indeed, implicit acquisition of a motor skill leads to higher retention than explicit acquisition [36]. During walking, the implicit adaptation of step length leads to higher aftereffects [37]. However, our participants demonstrated opposite effects from what would be expected if they had implicitly learned step position when given temporal feedback; we noted reduced aftereffects in step position, not increased aftereffects. Alternatively, participants may have relied on explicit, voluntary step timing control to achieve step position, and when that explicit feedback disappeared, so too did the voluntary step position strategy. In this case, the effect of asymmetric temporal feedback on step position may not carry over into subsequent walking conditions unless feedback is continually provided.

This study is not without limitations. There was a discrepancy in target accuracy between groups, where the Temporal Feedback group stepped on-target more frequently than the Spatial Feedback group (86% ± 9% compared to 77% ± 7%). Although the mean difference was only 9.4% of the total steps taken (113 steps of 1200 steps), the difference between group accuracy might influence results. Additionally, the chosen step timing and step position targets may not truly reflect the desired “end goal” of the fast and slow legs. However, there were no differences in step timing or position adaptation plateaus between the No Feedback group and the Feedback groups. Therefore, it appears that the chosen targets led participants to a similar final walking pattern as they implicitly would reach walking 1200 steps with no feedback. Although our sample size is at the higher end of similar studies on the effects of visual feedback on gait adaptation, it is still relatively small and that may affect our results. Finally, although there was a consistent number of researchers involved in every data collection, there were up to four uninvolved researchers present during some data collections, processing data. We do not believe these uninvolved researchers influenced the data. However, as there is no current understanding of the effect of non-observers on gait, it is possible these uninvolved researchers influenced our study.

## Conclusions

Healthy young adults can respond to asymmetric temporal and spatial feedback during gait adaptation without altering step length difference, step timing asymmetry, and step position asymmetry. Walking with asymmetric temporal feedback results in smaller aftereffects in step position compared to walking without feedback, supporting the idea that temporal gait adaptation may not be manipulated without also affecting spatial adaptation.

## Supporting information

S1 Data (XLSX)
